# The driving forces behind virtual water reduction in the Yangtze river delta urban agglomeration

**DOI:** 10.1016/j.isci.2025.114253

**Published:** 2025-11-27

**Authors:** Linmei Zhang, Yongxi Ma

**Affiliations:** 1 School of Economics and Management, Zhejiang Sci-Tech University, Hangzhou 310018, China; 2 Zhejiang Academy of Eco-Civilization, Zhejiang Sci-Tech University, Hangzhou 310018, China

**Keywords:** Earth sciences, Environmental science, Natural resources, Hydrology

## Abstract

Virtual water trade and regional specialization can enhance water security, yet their drivers and impacts remain unquantified. In this study, we propose a system-based modeling framework to trace virtual water transfer patterns, identify intercity network relationships, and quantify the primary socioeconomic factors driving water footprint dynamics in the Yangtze river delta urban agglomeration. Our analysis reveals that the total water footprint of this region decreased by 6% between 2010 and 2020. Water-scarce cities such as Yancheng, Yangzhou, and Tai'zhou in Jiangsu province serve as major production centers for water-intensive goods, accounting for nearly half of the region’s total virtual water exports. Changes in water use intensity, production structure, and final demand composition are identified as critical contributors to water footprint reductions in cities across Zhejiang and Anhui provinces. These findings underscore the necessity of cross-regional cooperation to address inefficient water resource allocation and mitigate unintended increases in water stress.

## Highlights

• The total water footprint of the YRDUA decreased by 6% over 2010–2020
• Yancheng, Yangzhou, and Tai'zhou are the net virtual water outflow areas
• Per capita demand and population growth are key water footprint drivers
• Targeted regulation needed for rising water footprints in Jiangsu cities

## Graphical abstract

**Figure:**
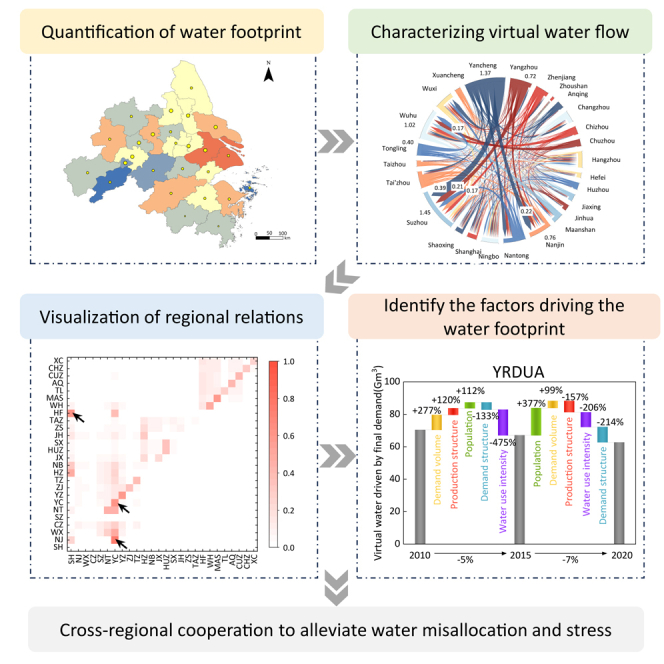

## Introduction

As a fundamental natural resource and strategic economic resource, the growing disparity between the uneven spatial distribution of water resources and regional development demands has become increasingly pronounced.^1^^,^^2^ Virtual water, as an invisible water resource embedded in goods and services, achieves spatial redistribution through inter-regional trade, offering an approach to mitigate the contradiction between water supply and demand.^3^^,^^4^^,^^5^ With the acceleration of urbanization and deepening of regional division of labor, the governance model for virtual water trade is transitioning from single administrative jurisdiction management to regional collaborative governance.^6^^,^^7^ Particularly in highly integrated regions in China, the scale of cross-administrative virtual water flow continues to expand, rendering the traditional territorial-based water resources governance system inadequate to address this emerging challenge.^8^^,^^9^

The implementation of the national strategy for the integrated development of the Yangtze river delta urban agglomeration (YRDUA) provides an ideal case study for examining the collaborative governance mechanism of virtual water transfer. Located on the southeast coast of China, the YRDUA encompasses 26 cities across four provinces (e.g., Shanghai, Jiangsu, Zhejiang, and Anhui). As one of the most economically developed and populous regions in China, it generates 20% of the country’s GDP while utilizing only 4% of its water resources.^10^^,^^11^ Despite relatively high-water resource utilization efficiency, the issue of regional water resource misallocation remains severe.^12^ In recent years, virtual water flows within the region have exhibited characteristics of networking and multi-center dynamics, posing significant challenges to the traditional administrative-region-based water resources management model.^13^ Consequently, elucidating the spatiotemporal characteristics and driving mechanisms of virtual water transfer among cities holds substantial theoretical and practical significance for optimizing water resource collaborative governance in the YRDUA and supporting high-quality regional development.

However, there remains a notable absence of a systematic approach or framework that effectively reveals sustainable and efficient water resource management strategies within the context of regional integration. While several studies have examined virtual water at the scale of urban agglomerations by constructing multi-regional input-output (MRIO) tables^2^^,^^14^^,^^15^ or life cycle assessment,^16^^,^^17^^,^^18^ there is a limited understanding of dynamics change and driving factors of these virtual water flows in terms of time and space (Table S1). Analyzing the Yangtze river delta as an interconnected system allows us to uncover the net virtual water transfers and dependencies between its core and peripheral cities, offering insights for regional policy coordination that city-level studies cannot provide. As a result, we currently lack deep insights into the socioeconomic factors driving changes in virtual water flows within highly interconnected and economically intensive urban agglomerations. Such information is crucial for identifying regional water stress mitigation options caused by water resource misallocation from a supply-chain perspective and enhancing water use efficiency.

Input-output analysis (IOA) has been widely applied to address environmental issues associated with economic activities, such as carbon emissions,^19^^,^^20^ air pollution,^21^^,^^22^ water resources,^2^^,^^13^ energy consumption,^8^^,^^23^ and land use.^24^^,^^25^ Network control analysis (NCA), a tool derived from ecological network analysis, has been employed to determine the dominance of one network component over another, which can effectively reveal intercomponent relationships and identify the key processes in water flow,^26^ energy flow,^27^^,^^28^ carbon flow,^29^^,^^30^ and energy-water-carbon flow.^31^^,^^32^ Additionally, structural decomposition analysis (SDA) has been recognized for assessing the influence of socioeconomic factors on water footprint,^33^^,^^34^ energy footprint,^23^^,^^35^ and carbon footprint.^29^^,^^30^^,^^36^ The integration of these approaches could provide significant insights into the structure and pathways of virtual water transfer between cities, revealing the opportunities of improving the efficient management of water resources through supply chain regulation.

In this study, we evaluate the transfer characteristics of virtual water and driving factors at the urban agglomeration scale from 2010 to 2020 using a system-based tracking framework that integrates IOA, NCA, and SDA. We calculate the water footprint of the YRDUA using a city-level input-output model, accounting for variations in production efficiencies and economic structures across cities. Based on this, we track virtual water flows embodied in inter-regional trade. Subsequently, we identify network relationships among cities, emphasizing the predominant influence of one city over another in directing virtual water transfer. Finally, we determine the key drivers behind the expansion of the water footprint over time. Through this analysis, we highlight critical strategies for alleviating water shortages and improving collaborative water resource management efficiency through a whole supply-chain perspective.

## Results

### Spatiotemporal variations of water footprint

**Figure 1:**
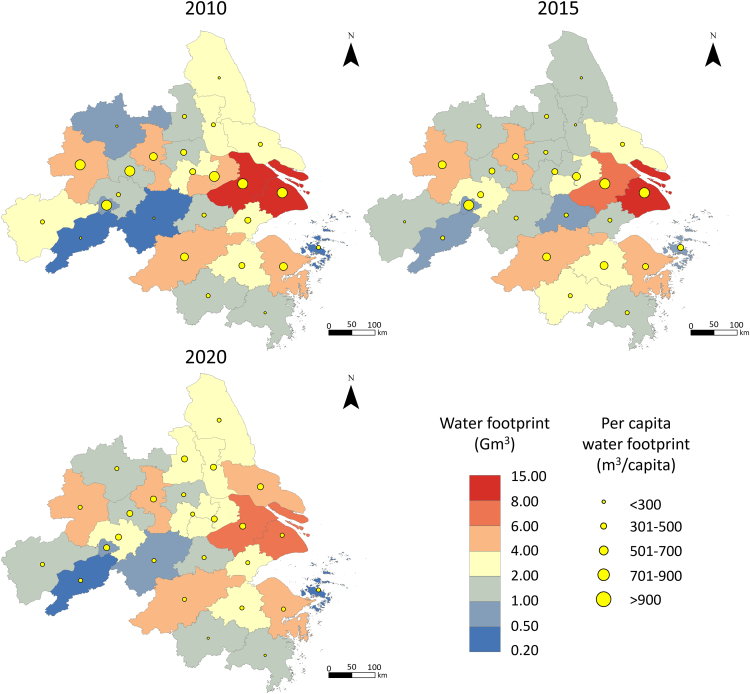
Changes of water footprint in the YRDUA over 2010–2020

Both the water footprint and the per capita water footprint of the YRDUA exhibited a downward trend from 2010 to 2020 (Figure 1). Our findings indicate that total water footprint driven by final demand in the YRDUA decreased by 6% (from 76 to 72 Gm^3^) during this period, which we primarily attribute to the implementation of stringent water conservation policies, most notably China’s “three red lines” policy. This decline can be deconstructed into several policy-driven mechanisms, such as industrial efficiency and restructuring, agricultural water-saving technologies, and urban water demand management. As of 2020, the top five cities with the highest water footprint were Shanghai, Suzhou, Hangzhou, Nanjing, and Hefei, all exceeding 4.5 Gm^3^, collectively accounting for nearly 42% of the total water footprint. Conversely, Tongliang, Chizhou, Xuancheng, and Zhoushan, had the smallest water footprint, each below 1.0 Gm^3^. Additionally, Shanghai achieved the largest reduction in its water footprint, decreasing by 46% (from 14.7 to 7.9 Gm^3^), mainly due to tightening water use constraints and decreasing water intensity. This was followed by Anqing (43%), Ma’anshan (27%), and Tongling (20%). Cities such as Wuxi, Suzhou, Zhenjiang, Jinhua, Taizhou (in Zhejiang province), and Hefei also experienced reductions in their water footprints ranging from 4% to 18%. However, during this period, the water footprint in the remaining cities of the YRDUA increased to varying extents. Notably, Wuhu, Chizhou, and Xuancheng in Anhui province witnessed the largest growth rates (91%–172%), while Nanjing, Changzhou, Nantong, Yangzhou, and Tai'zhou in Jiangsu province experienced growth rates of 14%–44%. Cities such as Hangzhou and Ningbo in Zhejiang province saw increases of less than 10%.

Overall, per capita water footprints of the YRDUA were 606, 560, and 440 m^3^/capita in 2010, 2015, and 2020, respectively. Cities such as Suzhou, Tai'zhou, and Yangzhou in Jiangsu province were identified as having the largest per capita water footprints (579–588 m^3^/capita) in 2020, approximately twice that of cities like Jinhua, Taizhou (in Zhejiang province), and Anqing (ranging from 228 to 312 m^3^/capita). From 2010 to 2020, the per-capita water footprint in most cities demonstrated a general decreasing trend, with reductions ranging from 10% to 69% across different cities. Only eight cities exhibited an increasing trend, with the most significant increases observed in Xuancheng (203%), Chizhou (149%), and Chuzhou (53%). Despite having the largest total water footprint, Shanghai maintained a relatively low per capita water footprint in 2020. In that year, Shanghai’s per capita water footprint was 319 m^3^/capita, lower than the average level of the YRDUA, and 69% less than in 2010. Similarly, although cities such as Suzhou, Ma’anshan, and Tongling had a high per capita water footprint in 2020, they had decreased significantly by 57%, 56%, and 55%, respectively, compared to 2010 levels (exceed 1,100 m^3^/capita). The decrease in the per capita water footprint is particularly significant given the concurrent pressures of a rapidly expanded population and a constrained total volume of available water resources. This achievement underscores the effectiveness of the implemented policies in promoting water-saving technologies and managing demand, leading to a reduction in water use even as the population served increased.

Further sector-level decomposition shows that Agriculture is responsible for ∼60% of total virtual water in the YRDUA, followed by production of electric power (13%–19%) and production of gas and water (∼10%) over 2010–2020 (Figure S1). There is a considerably high demand for the imports of agricultural products like soybeans, vegetables, and sea food, due to the dense population and higher consumption level in cities such as Shanghai, Suzhou, and Hangzhou. It highlights the inextricable link between food security and water security in the YRDUA and suggests that optimizing regional agricultural trade patterns and local sustainable agricultural practices should be a dual priority in water resources management policy. Meanwhile, a large amount of electricity and water are consumed to meet the needs of industrial production activities, such as the production of textiles, clothing, furniture and wood products, heavy machinery, precision machinery, and non-ferrous metals. Enhancing water-use efficiency in these sectors, therefore, requires a synergistic approach that combines industrial water-saving technologies with improvements in the energy efficiency of water systems, moving beyond siloed management.

### Virtual water-flow networks

**Figure 2:**
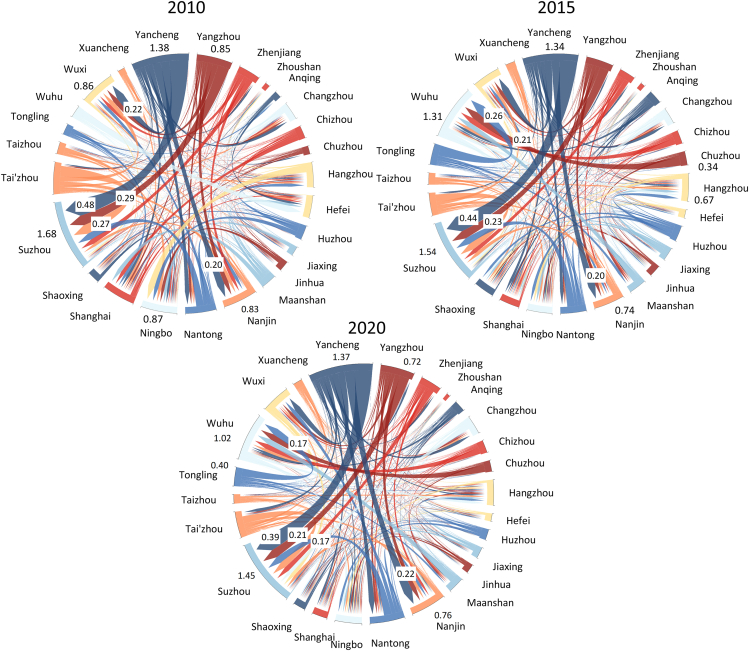
Net transfer of virtual water flow among cities in the YRDUA over 2010–2020 (Unit, Gm^3^/year)

We show the changes of virtual water flow across cities in the YRDUA over 2010–2020 (Figure 2). The total amount of virtual water transfer within the YRDUA has shown a downward trend, dropping from14 Gm^3^ in 2010 to 12 Gm^3^ in 2020, with a decline rate of 14%. It is worth noting that the decline from 2015 to 2020 showed an accelerating feature, with a decline rate of 11%. Among the 26 cities analyzed, Suzhou, Yancheng, and Ningbo ranked as the top three cities with the highest virtual water volumes in 2010. After 2015, Ningbo’s virtual water volume began to decline, stabilizing at approximately 0.5 Gm^3^, whereas Hefei’s virtual water volume gradually increased, surpassing 0.9 Gm^3^.

The top five cities in terms of net virtual water imports were Suzhou, Ningbo, Hefei, Nanjing, and Wuxi, all exceeding 0.5 Gm^3^, collectively accounting for roughly two-thirds of the total virtual water imports of the YRDUA during this decade. This concentration underscores their roles as major consumption hubs and economic powerhouses with dense populations and advanced manufacturing or service sectors that outstrip their local water resource capacity. Conversely, Yancheng, Yangzhou, Tai'zhou, and Nantong in Jiangsu province served as the primary producers, contributing nearly half of the total virtual water exports. This creates significant water stress, with these cities exhibiting water scarcity indices 1.2–2.5 times higher than regional averages.

The major export-import pairs with significant flows were predominantly within Jiangsu province, including Yancheng-Suzhou, Yangzhou-Suzhou, and Tai'zhou-Suzhou over 2010–2020. Notably, Suzhou emerged as the largest virtual water importer, with a total inflow of 1.64 Gm^3^ in 2010, primarily sourced from high water-stress cities such as Yancheng (0.48 Gm^3^) and Yangzhou (0.29 Gm^3^). This trend persisted even after 2015. Over this decade, Hangzhou became an enhanced virtual water consumer, with its net virtual water imports increasing from 0.37 Gm^3^ in 2010 to 0.44 Gm^3^ in 2015, representing a 19% growth. Shanghai demonstrated an opposite trajectory, with its net virtual water imports declining from 0.63 Gm^3^ to 0.46 Gm^3^, a reduction of 28%. The most dramatic transformation occurred in Changzhou, which completely reversed its virtual water role from a net importer (0.27 Gm^3^ in 2010) to a net exporter (0.20 Gm^3^ in 2015). The latest virtual water transfer data show that Changzhou is willing to produce water-intensive products, such as grain, leading to more such virtual water outflows. This transformation requires Changzhou to strike a delicate balance between specialized production and alleviating local water resource stress.

Detailed analysis reveals that Figure S2 illustrates the transfer characteristics of virtual water trade within the YRDUA at the sectoral level, considering both production and demand perspectives over the period 2010–2020. During this decade, the virtual water associated with the manufacturing of clothing, accommodation, and catering, and administration of water, environment, and public facilities increased by 5%, 8%, and 12%, respectively. Conversely, the virtual water in the remaining sectors decreased to varying extents, ranging from 1% to 45%. Notably, the virtual water in the production of electric power decreased by 45%, primarily attributed to the transition of the power structure from water-intensive methods to wind-power generation. The overall pattern of virtual water trade was predominantly influenced by agriculture, production of electric power, and production of gas and water, collectively accounting for approximately 83% of the total virtual water trade during this period. By 2020, Yancheng and Tai'zhou in Jiangsu province emerged as the most significant exporters of agriculture-related virtual water, contributing 19% of the outflow, whereas Suzhou and Shanghai were identified as the primary importers, representing 20% of the inflow. Additionally, Shanghai and Suzhou accounted for 39% of the total virtual water induced by the final demand of electric power.

**Figure 3:**
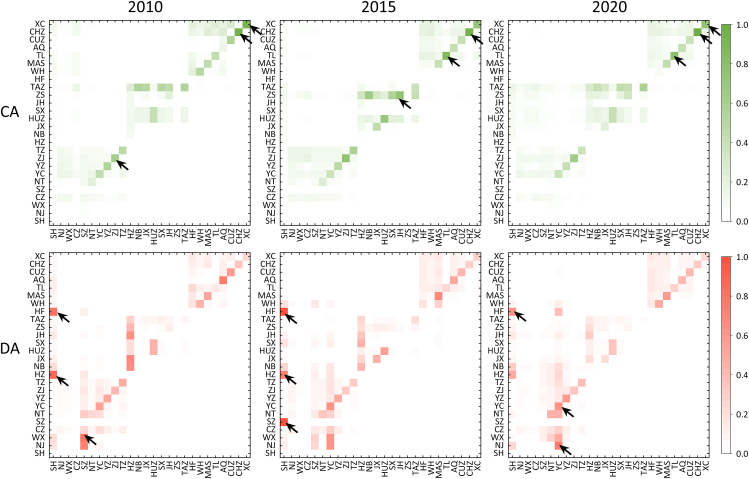
Control and dependence of virtual water flow exchange across cities in the YRDUA over 2010–2020 Arrows points to the strongest control of inter-regional relationships. Notes: Shanghai (SH), Nanjing (NJ), Wuxi (WX), Changzhou (CZ), Suzhou (SZ), Nantong (NT), Yancheng (YC), Yangzhou (YZ), Zhenjiang (ZJ), Tai'zhou in Jiangsu province (TZ), Hangzhou (HZ), Ningbo (NB), Jiaxing (JX), Huzhou (HUZ), Shaoxing (SX), Jinhua (JH), Zhoushan (ZS), Taizhou in Zhejiang province (TAZ), Hefei (HF), Wuhu (WH), Ma’anshan (MAS), Tongling (TL), Anqing (AQ), Chuzhou (CUZ), Chizhou (CHZ), and Xuancheng (XC).

The control and dependence relationships between cities of virtual water flow networks over 2010–2020 are shown in Figure 3, which disclose the potential mechanism for efficient water resource management. We find that the distribution of control is concentrated within the cities in Jiangsu and Anhui provinces, while the inter-city influence is more significant in Zhejiang province. For instance, the control degree of Zhenjiang, Chizhou, and Xuancheng over themselves has been evident over 2010–2020. In comparison, Ningbo exerted strong control over Taizhou in 2010, but gradually weakened after 2015. Furthermore, it can be observed that the control degree of Jinhua over Zhoushan was prominent in 2015, but experienced a significant decrease by 53% in 2020. This is mainly because the regional cooperation mode shifted from product dependence to industrial-chain coordination.

From the perspective of the recipient, while the overall pattern of interdependence among cities has remained relatively stable over the past decade, the degree of interdependence has undergone significant changes. For instance, post-2015, Hangzhou and Hefei notably reduced their reliance on Shanghai, gradually shifting their focus to Yancheng instead. Furthermore, Nanjing and Wuxi exhibited a dependency on Suzhou exceeding 70% in 2010, which diminished entirely by 2020. By contrast, their dependence on Yancheng reached 76% and 49%, respectively, by 2020. This trend suggests that the continuous evolution of regional industrial structures in recent years has led to water-intensive products such as agricultural produce and electronic goods, consumed in economically vibrant regions, being predominantly manufactured in Yancheng. Additionally, Ningbo, Jiaxing, and Jinhua demonstrated a dependency on Hangzhou exceeding 65% in 2010, but this decreased after 2015. Subsequently, these cities have progressively increased their reliance on other urban centers, including Shanghai, Yancheng, and Nantong.

### Socioeconomic drivers of water footprints

**Figure 4:**
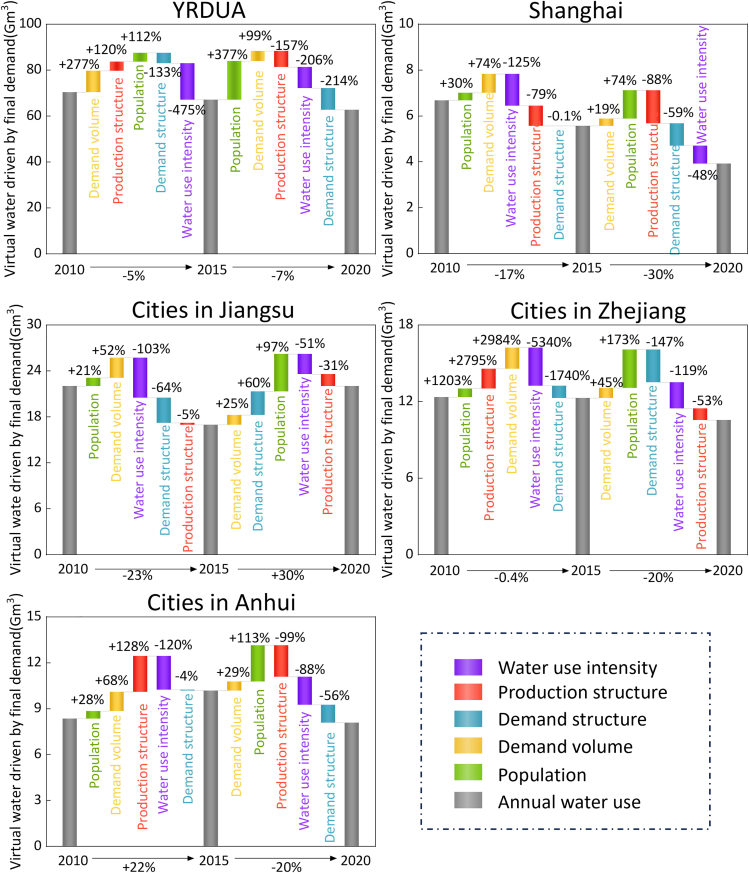
Contributions of socioeconomic factors to final demand-driven virtual water in the YRDUA over 2010–2020

Overall, our analysis reveals that changes in per capita demand volume and population were the primary drivers of the increase in water footprint in the YRDUA between 2010 and 2020. However, these effects could be mitigated by improvements in water use intensity, adjustments in the economic production structure, and shifts in the final demand structure (as shown in Figure 4). During 2010–2015, the combined influence of the economic production structure, per capita demand volume, and population growth resulted in a 509% increase in water footprint. This was partially offset by enhancements in water use efficiency (−475%) and the upgrading of the final demand structure (−133%), leading to an overall 5% reduction in water footprint during this period. From 2015 to 2020, the contribution of production structure optimization to water footprint reduction became increasingly significant, particularly in Zhejiang and Anhui provinces. Although the impact of per capita demand volume and population remained substantial, the other three factors largely counteracted their effects, resulting in respective decreases of 14% and 20% in the water footprints of these two provinces. Conversely, changes in the final demand structure caused a 30% increase in the water footprint of Jiangsu province during this period, primarily due to the rising demand for high-water-consuming industries.

A critical challenge that warrants attention is the substantial variation in the contribution of socioeconomic factors to the water footprint among cities during 2010–2020. Technological advancements resulted in a reduction exceeding 1.3 Gm^3^ in the water footprints of Shanghai, Suzhou, and Hangzhou between 2010 and 2015. However, the contribution of technological advancements to reducing water footprints exhibited a declining trend in most cities over 2015–2020. By 2020, technological advancements contributed to only a 0.5–0.8 Gm^3^ reduction in the water footprints of these three cities. The variation in economic production structures emerged as another significant factor influencing water footprint reductions from 2015 to 2020, particularly for cities in Zhejiang and Anhui provinces. For instance, in cities such as Hefei, Wuhu, and Hangzhou, changes in production structure led to an increase in water footprints by more than 0.4–0.6 Gm^3^ during 2010–2015. Subsequently, through optimizing production structures, their water footprints decreased by 24%, 14%, and 11%, respectively, over the following five years. The varying trajectories among cities call for differentiated and context-specific policies: mature megacities like Shanghai may need to focus on ultra-high efficiency and circular economy systems, while developing hubs like Hefei must embed water scarcity as a core criterion in their industrial planning from the outset to avoid locking into a water-intensive development path.

**Figure 5:**
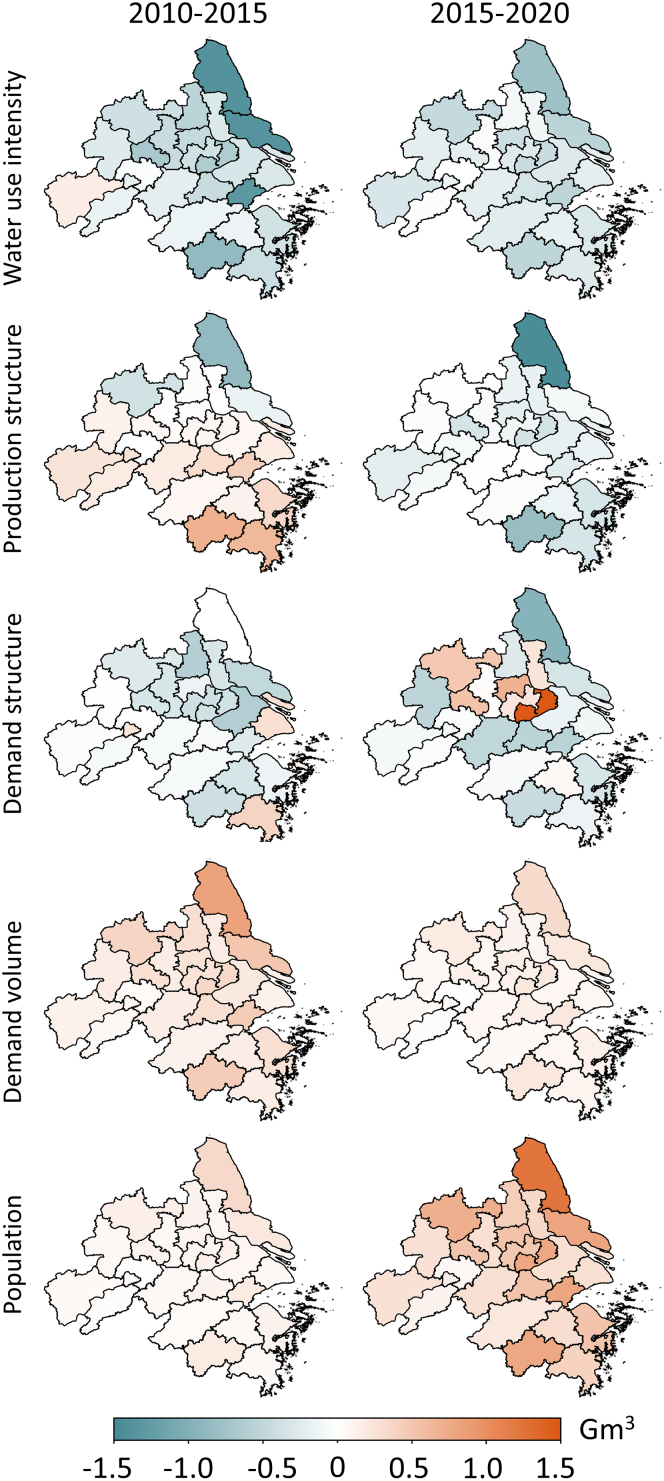
Contributions of socioeconomic factors to final demand-driven virtual water cross cities over 2010–2020

Nevertheless, a notable challenge in reducing water footprints is the overall increase in water footprints in cities located in Jiangsu province due to shifts in final demand structures. For example, in cities such as Nantong, Yangzhou, and Nanjing, changes in demand structure reduced water footprints by 0.2–0.4 Gm^3^ during 2010–2015 (Figure 5). However, due to alterations in demand structure in the subsequent five years, water footprints in these cities increased by 73%, 42%, and 37%, respectively. This underscores the significant variability in pathways toward rapid development depending on regional expansion of high water-consuming industries such as textiles, chemicals, and power. Meanwhile, changes in final demand volume and population growth collectively contributed to overall increases in water footprints. Notably, the contribution of population growth to water footprint increases has become increasingly significant, particularly in cities such as Yancheng, Jiaxing, and Jinhua, where population growth caused water footprints to rise by around 0.3 Gm^3^ during 2015–2020.

The sectoral decomposition of driving factors within the YRDUA indicates that changes in water use intensity and production structure have distinct impacts on efforts to reduce the water footprint (Figure S3). The construction, administration of water, environment and public facilities, and healthcare and social-work sectors were the only ones that experienced the most significant increases in water footprint between 2010 and 2015. In the construction sector, despite a notable decline in water use intensity (−70%), the increase in production structure (57%) and final demand structure (56%) contributed to a 3.8 Gm^3^ rise in water footprint. Similarly, although water use intensity decreased, an expansion in production structure, demand structure, per capita demand, and population resulted in a 28% increase in water footprint for both the administration of water, environment, and public facilities, and health care and social work sectors. However, from 2015 to 2020, the water footprints of sectors such as manufacturing of measuring instruments, manufacturing of food, manufacturing of clothing, and manufacturing of paper exhibited upward trends and require targeted regulatory attention. For example, driven by continuous population growth, rising per capita demand, and shifts in final demand structure, the water footprints of the manufacturing of measuring instruments and manufacturing of paper sectors increased by 109% and 70%, respectively, during this period.

## Discussion

The goal of achieving sustainable development of regional integrated water resources entails the exploration of water-shortage alleviation pathways from the supply-chains perspective.^37^^,^^38^^,^^39^ Although many studies have provided valuable insights into the changes in water footprints and virtual water transfer, they are often hindered by the lack of the latest high-quality data, thus not focusing on the collaborative management of water resources among cities in the context of regional integration development.^14^^,^^40^ In contrast, our study leverages detailed and recent datasets to overcome this limitation. Grasping the impact of local water resources, the spatiotemporal pattern of virtual water transfer across cities, and its driving factors is crucial for alleviating water resource shortages caused by final demand and ensuring the sustainability of water resources and the entire economic system.

In this study, both the total water footprint and per capita water footprint of the YRDUA exhibited a declining trend from 2010 to 2020. However, the disparities among cities have progressively widened, reflecting an uneven distribution of urban coordinated development capabilities and an inequitable allocation of water conservation responsibilities. Recently, Shanghai, Jiangsu, Zhejiang, and Anhui jointly issued the “Action Declaration on Improving Water Efficiency in the Yangtze River Delta,” which outlines that by 2027, water consumption per 10,000 yuan of GDP and per 10,000 yuan of industrial added value will each decrease by more than 20% compared to 2020 levels. This indicates that the region’s water footprint is expected to decline further in the future. Effectively managing limited water resources to support the high-quality development of various cities has thus become critically important.^13^^,^^41^ In addition to enhancing water-saving measures at the point of consumption, it is also essential to regulate virtual water flows through trade networks as part of integrated regional water-resource management strategies. Interregional trade often results in the transfer of virtual water from water-scarce regions to water-abundant ones, potentially exacerbating water stress in arid areas.^40^^,^^42^ Therefore, there is an urgent need to establish a cross-city ecological compensation mechanism that leverages economic instruments and institutional design to address the imbalance between rights and responsibilities in regional water governance.

Within urban agglomerations, cities should not only pursue internal system optimization but also prioritize regional coordinated development. In water-scarce regions such as Jiangsu province, the active promotion of advanced water-saving technologies and closed-loop water recycling systems is essential. Among the 17 cities with net virtual water outflows, nine cities (Yancheng, Nantong, Yangzhou, Tai'zhou in Jiangsu Province, Changzhou, Zhenjiang, Jiaxing, Ma’anshan, and Wuhu) exhibited a water stress index (WSI)> 0.4 in 2020, indicating a significant level of water scarcity. Notably, the dependence of Nanjing, Wuxi, and other cities on Yancheng is progressively increasing, potentially elevating Yancheng’s water stress from high to extremely high levels in the future. This pattern of virtual water trade poses a threat to water security due to substantial losses of local virtual water resources, compounded by their limited water resource endowments. A more strategic and differentiated approach should therefore be adopted, leveraging the comparative advantages of each region. Specifically, Jiangsu province could be encouraged to focus on high-value-added, low-water-intensity sectors such as research and development and headquarters economies. Meanwhile, regions with relatively lower water stress (WSI <0.3)—including Huzhou, Jinhua, and Taizhou in Zhejiang province, and Chuzhou, Xuancheng, Anqing, and Chizhou in Anhui province—could be supported in developing water-intensive industries, provided they adhere strictly to the highest water efficiency standards and ecological redlines, and are integrated into robust virtual water trade frameworks.^1^^,^^43^ Conversely, among the remaining nine cities with net virtual water inflows, only three cities (Hangzhou, Ningbo, and Shaoxing) maintained a water-sufficient status, characterized by a WSI <0.26. Despite their relatively abundant water resources, the growing population and rapid economic development in these cities have driven local final demands, which are largely met by exploiting water resources from other cities. This dynamic exacerbates the risk of water scarcity in some water-stressed cities where water-intensive productions are concentrated.

From a production perspective, reducing water use intensity and optimizing the economic production structure remain the most effective strategies for alleviating water shortages in the YRDUA. However, the contribution of technological advancements to reducing water footprints has shown a declining trend in most cities. This necessitates further dynamic adjustments in upstream and midstream cities with water-intensive and labor-intensive industries, such as agriculture and manufacturing, to enhance the potential for mitigating water scarcity.^9^^,^^44^ Based on the analysis of virtual water transfer sectors in this study, formulating differentiated water transfer strategies requires proposing solutions that align with the unique industrial characteristics of each city. Strategies should be tailored to the specific industrial structures, resource endowments, infrastructure, and functional positioning of each city, aiming to achieve greater efficiency with fewer resources.^8^^,^^45^ For example, cities such as Yancheng, Shanghai, and Yangzhou should focus on optimizing their agricultural structures, as well as develop and promote advanced artificial intelligence algorithms for efficient management of agricultural production activities. Additionally, Shanghai needs to further optimize its energy structure by gradually transitioning thermal power generation to offshore wind and solar power generation, thereby reducing water use associated with thermal power generation.

From a consumption perspective, changes in the final demand structure should be closely monitored due to the complex role they play in alleviating water stress, particularly for cities located in Jiangsu province. Between 2015 and 2020, the demand for high-water-consuming industries—such as agriculture and the textile industry—increased in cities like Nantong, Yangzhou, and Tai'zhou in Jiangsu province, thereby expanding their water footprint. Moreover, with rising disposable incomes among residents, consumption patterns have continued to upgrade. The growing demand for meat, eggs, dairy products, processed foods, seafood, buildings, and related services has led to a significant increase in virtual water imports to meet domestic needs.^46^^,^^47^ For example, the construction sector experienced a sharp rise in virtual water inflows, highlighting the necessity of optimizing building material selection and supply chain management. This can be achieved through the use of recycled materials, promoting water-efficient alternatives such as cementitious materials, bamboo, and engineered wood products, developing resource-efficient construction techniques and technologies, and encouraging the adoption of end-use water-saving appliances. Conversely, for other cities where water footprints may remain stable or even decline, water stress can be further mitigated by fostering low-water-consuming industries such as information technology, research and development, and high-end manufacturing—including certain electronics and precision instruments.

### Limitations of the study

It is acknowledged that the present study has certain limitations that warrant consideration in future research. First, the analysis primarily focuses on the blue water footprint, largely due to the unavailability of comprehensive data required for accurately assessing green and gray water footprints. The omission of green and gray water components may lead to an underestimation of agricultural water consumption and does not account for water quality degradation, thereby limiting the comprehensiveness of the total water footprint assessment. Second, the use of MRIO tables for the years 2012, 2015, and 2017 introduces uncertainty into the estimation of results for the intervening period from 2010 to 2020. These limitations could be addressed in subsequent studies through the utilization of more accurate, consistent, and up-to-date datasets on water resources and economic transactions. It is recommended that future research pursue a more integrated assessment framework that incorporates blue, green, and gray water footprints. Such an approach would provide a more holistic understanding of human-induced pressures on freshwater systems and support the development of more effective and coordinated water resource management policies.

## Resource availability

### Lead contact

Further information and requests for resources should be directed to and will be fulfilled by the lead contact, Yongxi Ma (myx@zstu.edu.cn).

### Materials availability

This study did not generate new unique reagents.

### Data and code availability

• All the source data used in this analysis are listed in the key resources table.
• This paper does not report original code.
• Any additional information required to reanalyze the data reported in this paper is available from the lead contact upon request.

## Acknowledgments

This study was supported by 10.13039/100007219Zhejiang Provincial Natural Science Foundation of China under Grant No. QN25G030048, LZ25G030001, and the 10.13039/501100004731Science Foundation of Zhejiang Sci-Tech University under Grant No. 23092220-Y.

## Author contributions

Conceptualization, L.Z. and Y.M.; methodology and investigation, L.Z.; writing, reviewing, and editing, L.Z. and Y.M.; funding acquisition, L.Z. and Y.M.; supervision, Y.M. All authors approved and contributed to paper writing.

## Declaration of interests

The authors declare no competing interests.

## STAR★Methods

### Key resources table

**Table:** 

REAGENT or RESOURCE	SOURCE	IDENTIFIER
**Deposited data**		

Water use data	Water Resources Bulletins	https://slt.zj.gov.cn/art/2021/8/30/art_1229243017_4719972.html
Water use structure of industrial sub-sectors	2008 China Economic Census Yearbook	http://202.106.125.35/csydkns/navi/YearBook.aspx?id=N2010120121&floor=1
Population and GDP	China Urban Statistical Yearbook	https://www.stats.gov.cn/sj/ndsj/
China City-level MRIO Table	Carbon Emission Accounts and Datasets	https://www.ceads.net/data/input_output_tables/
Water stress index	Wang et al.^48^	https://wri.org.cn/sites/default/files/2022-01/baseline-water-stress-china.pdf

**Software and algorithms**		

MATLAB	The MathWorks	https://www.mathworks.com/products/matlab.html

### Experimental model and study participant details

Generally, in the study of water footprint, blue water, green water and grey water are mainly involved. The blue water footprint refers to the consumption of surface and groundwater, where consumption denotes the volume of water that is lost through evaporation or incorporated into a product. The green water footprint represents the volume of rainwater consumed during production processes, particularly relevant in agricultural and crop production contexts. The gray water footprint is defined as the volume of freshwater required to assimilate pollutants and maintain ambient water quality standards.^49^^,^^50^^,^^51^ However, considering data availability, regional trade impact and water resource regulatory policies, this study only takes into account the blue water footprint driven by production and consumption activities. We quantify the bule water footprint in the YRDUA based on sub-sectoral water use data and city-level MRIO tables over 2010-2020.

#### Water use in different sectors of the YRDUA

The data on agricultural, industrial, service, and residential water use, as well as the total available water resources (including surface water and groundwater) for 26 cities in the YRDUA were directly extracted from the Water Resources Bulletins of Shanghai, Jiangsu, Zhejiang, and Anhui provinces over the period 2010–2020. Given the absence of official statistics on sectoral water use at the city level, an indirect estimation method was employed to derive water use data for 42 sub-sectors across the 26 cities in the YRDUA. The detailed economic sector classification is provided in Table S2. The indirect estimation method is as follows:

(1)Agricultural water use data encompass farmland irrigation, forestry, animal husbandry, fishery, and aquaculture, which can be directly allocated to the corresponding economic sectors under *Agriculture.*
(2)Industrial sub-sector water use data were estimated based on detailed water use information from the 2008 China Economic Census Yearbook. It was assumed that the structure of industrial water use in Chinese provinces in 2010 remained largely consistent with that in 2008. Furthermore, the water use structure of industrial sub-sectors in each city was considered identical to that of its respective province. Using this approach, corresponding data for 2015 and 2020 were also obtained. The processed sub-industry data were subsequently assigned to relevant economic sectors, such as *Coal Mining and Dressing, and Petroleum and Natural Gas Extraction.*
(3)For the service sub-sector, water use data from 2010 to 2020 were estimated by referencing the water use structure derived from the 2008 China Economic Census Yearbook. These data were then distributed into corresponding economic sectors, such as *Wholesale and Retail Trade, Real Estate, and Healthcare and Social Work Activities.*

#### City-level MRIO tables used to construct water footprint accounting model

We obtained city-level MRIO tables in 2012, 2015 and 2017 (excluding Taiwan, Hong Kong and Macao) from Carbon Emission Accounts and Datasets.^52^ The table covers 313 regions with 42 sectors, including all 26 cities in the YRDUA, and other non-YRDUA provinces and municipalities of China.

To match the MRIO model and reflect the changes in virtual water flow during regional trade processes, the water usage data of cities other than the 26 cities in the YRDUA are also included. This means that the water usage for agriculture, industry and services in 313 regions has been quantified in the model and allocated to 42 sectors according to the above-mentioned method.

#### Other data

The data on population and GDP of cities in the YRDUA from 2010 to 2020 are sourced from the China Urban Statistical Yearbook. For example, we can assess the changes in per capita water footprint based on population data and water footprint data.

### Method details

#### Accounting water footprint of the YRDUA

Environmental input-output analysis, originally introduced by Leontief,^53^ has been widely applied to assess the comprehensive environmental impacts associated with economic activities across entire economies. In this study, we investigate the virtual water transfers embedded in interregional trade by constructing a YRDUA-MRIO model to quantify water footprints over the period from 2010 to 2020. The YRDUA-MRIO model enables the calculation of water footprints throughout the entire supply chain, encompassing both direct and indirect water consumption driven by regional final demand, as well as direct household water usage. Based on the principles of horizontal accounting equilibrium, the following formula can be derived:

Equation (1) X=AX+Y

where *A* stands for the direct consumption matrix; $X=\left(x_{i}^{s}\right)$ denotes the total output vector; $x_{i}^{s}$is the total output of sector *i* in city *s*; *Y* stands for the final demand vector.

Solving for X yields:

Equation (2) X=(I−A)−1×Y

where Leontief inverse matrix (*I*-*A*)^-1^ captures both direct and indirect inputs, indicating the demand of total output for satisfying one unit of final demand and *I* is the identity matrix.$A=\left[a_{ij}^{rs}\right]$, and aijrs=zijrsxjsis the intersectoral flows from sector *i* in city *r* to sector *j* in city *s*.

To assess the environmental impact and calculate the embodied water in the goods and services, the MRIO table is extended with coefficients for water use. The water footprint (WF) is calculated as

Equation (3) WF=e(I−A)−1Y+Hd

Where *e* stands for the water use per unit of economic output, *H*_*d*_ is household direct water consumption of city *s*. The WF is total water use associated with the production of goods and services along the entire supply chain plus household direct water use.

Then, the virtual water as depicted by the equation:

Equation (4) $$VW=\hat{e}{\left(I-A\right)}^{-1}\hat{Y}$$

where *VW* denotes virtual water of city *s*; $\hat{e}$ is a diagonalized column vector of water use intensity; $\hat{Y}$ is a diagonalized column vector of final demand.

#### Network analysis of virtual water flow

To analyze the virtual water flows among sectors within the trade network, this study employed the systemic analysis method known as Network Environ Analysis (NEA). Based on the step-by-step procedure proposed by Fath and Borrett (2006),^54^ we constructed virtual water flow networks for the YRDUA. The system balance of the virtual water flow network can be expressed as follows Fath and Borrett. The system balance of the virtual water flow network can thus be written as follows:

Equation (5) $$T_{i}^{in}=\sum_{j=1}^{n}f_{i,j}+z_{i},i=1,2,\ldots ,n$$

Equation (6) $$T_{j}^{out}=\sum_{i=1}^{n}f_{i,j}+y_{j},j=1,2,\ldots ,n$$

Equation (7) $$\text{TST}=\sum_{i=1}^{n}T_{i}^{in}=\sum_{j=1}^{n}T_{j}^{out}$$

where $T_{i}^{in}$ and $T_{j}^{out}$ denote the total inflow to and outflow from each urban component, respectively, respectively; *f*_*i*,*j*_ represents the virtual water flow from component *i* to *j*, *z*_*i*_ indicates the boundary inflows (i.e., external import) into component *i*, and *y*_*j*_ denotes the boundary outflows (i.e., export to other regions) from component *i*. The total system throughflow (TST) is calculated as the sum of throughflows across all 26 cities.

Subsequently, we applied the indicators of control allocation (CA) and dependence allocation (DA),^55^ to quantify the control and dependence relationships among cities concerning virtual water exchanges. Both CA and DA fall within the range of 0 to 1,and they are dimensionless. The larger the number, the higher the degree of control or dependence on other numbers. The values on the diagonal of the matrix represent the virtual water flow circulating within the city’s own economic system (e.g., the water embedded in goods and services produced and consumed within the same city). The CA and DA are derived from two pairwise integral flows *N* and *N*′.

Equation (8) $$N=\left(n_{i,j}\right)=\sum_{m=0}^{\infty }G^{m}=I+G^{1}+G^{2}+\ldots +G^{m}={\left(I-G\right)}^{-1}$$

Equation (9) $$N^{\prime }=\left(n_{i,j}^{\prime }\right)=\sum_{m=0}^{\infty }G^{\prime m}=I+G^{\prime 1}+G^{\prime 2}+\ldots +G^{\prime m}={\left(I-G^{\prime }\right)}^{-1}$$

Equation (10) $$G\left(n,n\right)=g_{i,j}=\frac{f_{i,j}}{T_{j}^{out}}$$

Equation (11) $$G^{\prime }\left(n,n\right)=g_{i,j}^{\prime }=\frac{f_{i,j}}{T_{i}^{in}}$$

where *N*=(*n*_*i*,*j*_) and $N^{\prime }=\left(n_{i,j}^{\prime }\right)$ are dimensionless integral matrices of metabolic flow, and *G*(*n*,*n*) and *G*′(*n*,*n*) are the corresponding direct dimensionless matrices of metabolic flow. *I* denotes the identity matrix. The self-feedback matrix *G*′^1^ represents flows between directly connected nodes (i.e., flows with a path length of 1), *G*′^2^ represents the flows of length 2 between nodes, and so forth. *g*_*i*,*j*_ and $g_{i,j}^{\prime }$ respectively denote nondimensional, output-oriented intercomponent flows from component *i* to component *j*, and input-oriented intercomponent flows from component *j* to component *i*, respectively.

(Equation 12) $$\text{CA}=\left[{ca}_{ij}\right]=\left\{ \begin{array}{c} n_{i,j}-n_{j,i}^{\prime }>0,{ca}_{ij}=\frac{n_{i,j}-n_{j,i}^{\prime }}{\sum_{i=1}^{n}\left(n_{i,j}-n_{j,i}^{\prime }\right)}\\ n_{i,j}-n_{j,i}^{\prime }\leq 0,{ca}_{ij}=0 \end{array}\right.$$

(Equation 13) $$\text{DA}=\left[{da}_{ij}\right]=\left\{ \begin{array}{c} n_{i,j}-n_{j,i}^{\prime }>0,{da}_{ij}=\frac{n_{i,j}-n_{j,i}^{\prime }}{\sum_{i=1}^{n}\left(n_{i,j}-n_{j,i}^{\prime }\right)}\\ n_{i,j}-n_{j,i}^{\prime }\leq 0,{da}_{ij}=0 \end{array}\right.$$

where 0 ≤ *da*_*i*,*j*_, *ca*_*i*,*j*_ ≤ 1, *ca*_*i*,*j*_ reflects the degree of control that compartment *j* exerts over compartment *i* based on the controller’s output environ, and *da*_*i*,*j*_ represents the degree of dependence of compartment *j* on compartment *i* from the observer’s input environ.

#### Assessment of water footprint drivers

Assessing the role of socioeconomic factors as drivers of the water footprint can provide valuable insights into addressing the mismatch in water resource distribution. Structural decomposition analysis (SDA) breaks down changes in economic or environmental variables into a set of explanatory factors that are closely associated with the input-output model.^56^ Analyzing the underlying drivers enables the identification of potential pathways for reducing the water footprint, thereby supporting informed policy-making. In this study, SDA was applied to examine the impact of various driving factors—such as technological change, economic system efficiency, structural change, population growth, and per capita demand—on the water footprint of the YRDUA. On the supply side, key drivers encompass technological change (measured by sector-specific direct resource intensity coefficients, such as water use per unit of output), economic system efficiency (reflected in changes in the Leontief inverse matrix, which indicate improvements in supply chain efficiency). On the demand side, the primary drivers are the structural change (referring to shifts in the composition of final demand, including transitions from water-intensive manufacturing to less water-intensive service sectors), population growth (directly derived from demographic data) and per capita demand (assessed through changes in final consumption per capita, reflecting variations in affluence and consumption patterns, such as rising meat consumption).

The study period from 2010 to 2020 was divided into two intervals: 2010–2015 and 2015–2020, to identify key factors influencing water footprint changes. The SDA model was formulated as follows:

Equation (14) WF=eLysyvp

where *WF* represents water footprint of the YRDUA. *e*, *L*, *y*_*s*_, *y*_*v*_ and *p* are water use intensity, economic production structure, final demand structure, final demand level (i.e., per capita final demand volume) and population, respectively. These contributions of factors can be further decomposed as:

Equation (15) ΔWF=ΔeLysyvp+eΔLysyvp+eLΔysyvp+eLysΔyvp+eLysyvΔp

where Δ*WF* rindicates the change in the water footprint of the YRDUA. Δ*e*, Δ*L*, Δ*y*_*s*_, Δ*y*_*v*_ and Δ*p* represent the changes in water footprint attributable to water use intensity, economic production structure, final demand structure, final demand level and population, respectively, while holding other factors constant.

Equation (16) $$\Delta eLy_{s}y_{v}p=\frac{1}{5}\left(e_{1}-e_{0}\right)L_{0}y_{s0}y_{v0}p_{0}+\frac{1}{20}\;\left(e_{1}-e_{0}\right)\left[L_{0}y_{s0}y_{v0}p_{1}+L_{0}y_{s0}y_{v1}p_{0}+L_{0}y_{s1}y_{v0}p_{0}+L_{1}y_{s0}y_{v0}p_{0}\right]+\frac{1}{30}\left(e_{1}-e_{0}\right)\left[L_{1}y_{s1}y_{v0}p_{0}+L_{0}y_{s0}y_{v1}p_{1}+L_{0}y_{s1}y_{v0}p_{1}+L_{1}y_{s0}y_{v1}p_{0}+L_{1}y_{s0}y_{v0}p_{1}+L_{0}y_{s1}y_{v1}p_{0}\right]+\frac{1}{20}\left(e_{1}-e_{0}\right)\left[L_{1}y_{s1}y_{v1}p_{0}+L_{1}y_{s1}y_{v0}p_{1}+L_{0}y_{s1}y_{v1}p_{1}+L_{1}y_{s0}y_{v1}p_{1}\right]+\frac{1}{5}\left(e_{1}-e_{0}\right)L_{1}y_{s1}y_{v1}p_{1}$$

The weighted average method proposed by Li (2005)^57^ was selected for its simplicity in decomposition; subscripts 0 and 1 denote the beginning and end years, respectively, in the time-level analysis. A similar calculation approach was applied to all other variables.

#### Measurement of variations in water stress

The degree of water scarcity is typically assessed using the Water Stress Index (WSI), defined as the ratio of annual water withdrawals—for domestic, industrial, and agricultural purposes—to the long-term average of renewable water resources.^58^^,^^59^^,^^60^ The WSI has been widely adopted to evaluate water availability at catchment or regional levels.^48^^,^^61^ Regional WSI values within the ranges of 0–0.1, 0.1–0.2, 0.2–0.4, 0.4–0.8, and above 0.8 correspond to low, low-to-medium, medium-to-high, high, and extremely high levels of water stress, respectively. The water stress for city *i* was calculated as follows:

Equation (17) $${WSI}_{i}=\frac{{WU}_{i}}{{RWR}_{i}}$$

where WU represents the volume of water supplied to end users, including losses during distribution, and is equivalent to total water withdrawals plus net physical water imports; RWR denotes the total renewable water resources—comprising surface water and groundwater—available within the city. Due to the unavailability of city-level data on net physical water transfers, local RWR was estimated under the assumption that cities primarily rely on locally available water sources to meet their demand.

#### Case study

We selected all 26 cities (including both urban and rural areas) within the YRDUA, China, as case studies to evaluate inter-regional virtual water transfers and their potential driving factors. Among these cities, Shanghai serves as the largest metropolis. Nine cities are located in Jiangsu Province (i.e., Nanjing, Wuxi, Changzhou, Suzhou, Nantong, Yancheng, Yangzhou, Zhenjiang, Taizhou), eight cities are situated in Zhejiang Province (i.e., Hangzhou, Ningbo, Jiaxing, Huzhou, Shaoxing, Jinhua, Zhoushan, Tai'zhou), and the remaining eight cities belong to Anhui Province (i.e., Hefei, Wuhu, Ma’anshan, Tongling, Anqing, Chuzhou, Chizhou, Xuancheng).

Although the total water resources in the YRDUA are more abundant than that in other regions, their spatial distribution is highly uneven, with per capita water availability amounting to only one-third of the national average. From 2010 to 2020, cities in Jiangsu Province experienced severe water stress, indicated by a water stress index exceeding 0.4. Meanwhile, water scarcity issues in other cities also intensified during this period (Figure S4). As of 2020, total water consumption across cities in the YRDUA ranged from 1.0 to 7.3 Gm^3^, with Shanghai, Yancheng, Nanjing, Nantong, and Suzhou ranking among the top 10% nationwide in terms of water usage volume. In highly urbanized cities such as Shanghai and Suzhou, manufacturing, service, and domestic water uses accounted for more than 78% of total water consumption, whereas in Yancheng, agricultural water use dominated, contributing to approximately 83% of its total water consumption.

To effectively address the imbalance between water supply and demand in the region, successive policy initiatives—such as the “Water Security Assurance Plan for the Integrated Development of the Yangtze River Delta Region” and the “Outline of the Integrated Development Plan for the Yangtze River Delta Region”—have emphasized the necessity of regional collaborative governance.

### Quantification and statistical analysis

Model Implementation: The structural decomposition analysis (SDA) was implemented and solved using MATLAB. All scripts are available in the Code Availability section.

Model Validation: The virtual water footprint calculated by our model was cross-validated against with other literature based on top-down water data.
