# Supplementary material for: The driving forces behind virtual water reduction in the Yangtze river delta urban agglomeration
Source: iScience. 2025 Nov 27;28(12):114253. doi: 10.1016/j.isci.2025.114253 (PMC12753269; doi:10.1016/j.isci.2025.114253)
Supplement: Document S1. Figures S1–S4 and Tables S1–S2 [file mmc1.pdf]

iScience, Volume *28*

Supplemental information

The driving forces behind virtual water reduction in the Yangtze river delta urban agglomeration

Linmei Zhang and Yongxi Ma

Supplementary Information

The Driving Forces Behind Virtual Water Reduction in the Yangtze River Delta Urban Agglomeration

Contents

(4 Figures, 2 Tables)

Figure S1. Contributions of economic sectors to virtual water of the YRDUA over 2010-2020.

Figure S2. Virtual water transfer changes between cities and sectors within the YRDUA over 2010-2020.

Figure S3. Contributions of different socioeconomic factors to water footprint at the sector level in the YRDUA over 2010-2020.

Figure S4. Changes of water stress index in the YRDUA over 2010–2020

Table S1. An overview of previous studies related to accounting of virtual water and water footprint.

Table S2. The aggregated and original sectors in the 2015 input-output table as an example.

Supplementary Figures

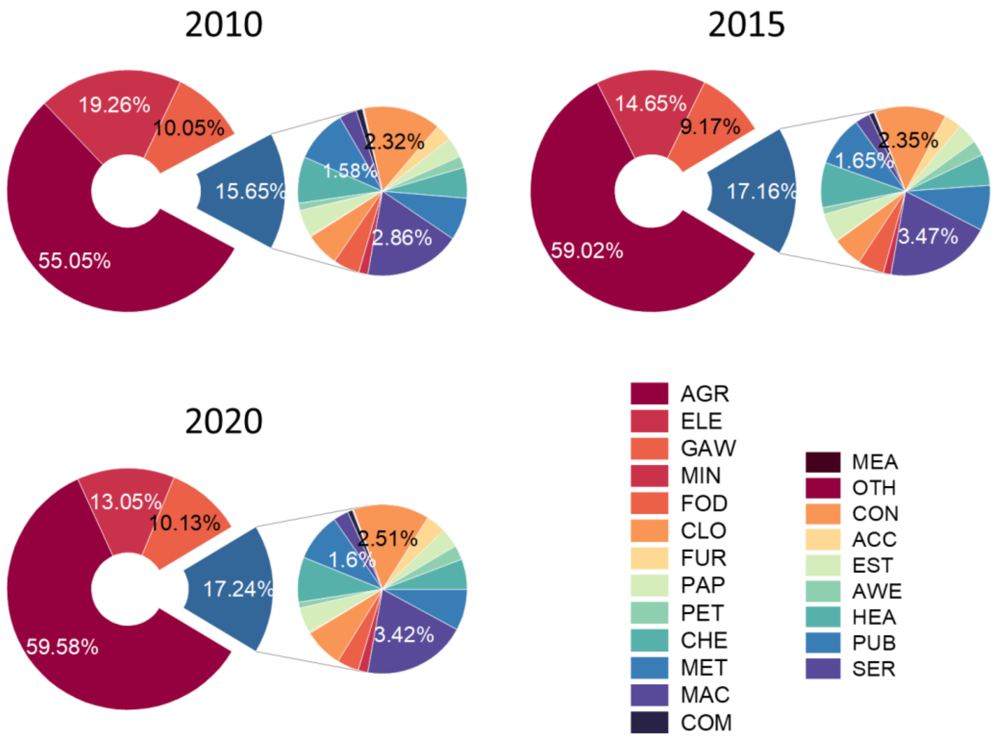

Figure S1. Contributions of economic sectors to virtual water of the YRDUA over 2010-2020

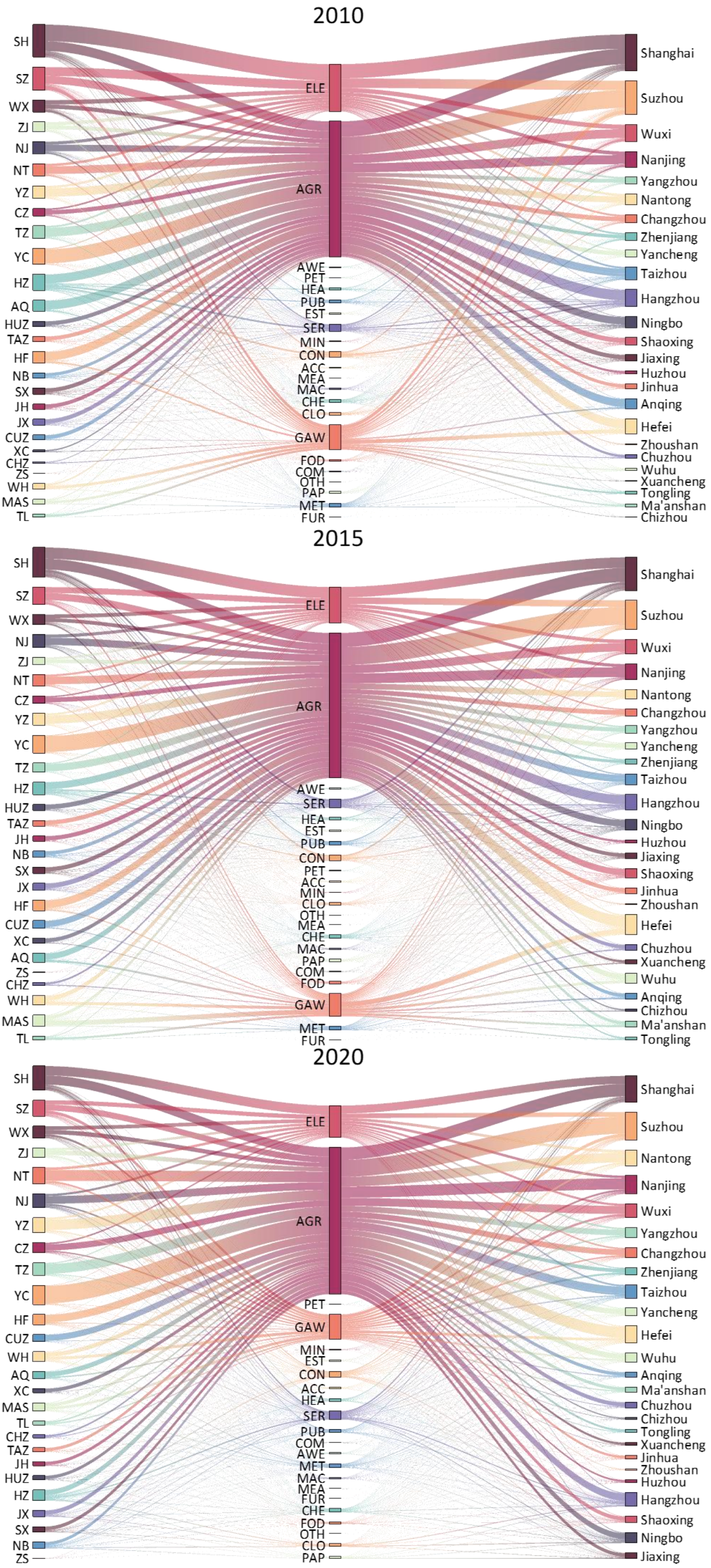
2010
SH
SZ
WX
ZJ
NJ
NT
YZ
CZ
TZ
YC
HZ
AQ
HUZ
TAZ
HF
NB
SX
JH
JX
CUZ
XC
CHZ
ZS
WH
MAS
TL
ELE
AGR
AWE
PET
HEA
PUB
EST
SER
MIN
CON
ACC
MEA
MAC
CHE
CLO
GAW
FOD
COM
OTH
PAP
MET
FUR
Shanghai
Suzhou
Wuxi
Nanjing
Yangzhou
Nantong
Changzhou
Zhenjiang
Yancheng
Taizhou
Hangzhou
Ningbo
Shaoxing
Jiaxing
Huzhou
Jinhua
Anqing
Hefei
Zhoushan
Chuzhou
Wuhu
Xuancheng
Tongling
Ma'anshan
Chizhou
2015
2020

Figure S2. Virtual water transfer changes between cities and sectors within the YRDUA over 2010-2020.

Notes: Shanghai (SH), Nanjing (NJ), Wuxi (WX), Changzhou (CZ), Suzhou (SZ), Nantong (NT), Yancheng (YC), Yangzhou (YZ), Zhenjiang (ZJ), Taizhou in Jiangsu province (TZ), Hangzhou (HZ), Ningbo (NB), Jiaxing (JX), Huzhou (HUZ), Shaoxing (SX), Jinhua (JH), Zhoushan (ZS), Taizhou in Zhejiang province (TAZ), Hefei (HF), Wuhu (WH), Ma'anshan (MAS), Tongling (TL), Anqing (AQ), Chuzhou (CUZ), Chizhou (CHZ), Xuancheng (XC).

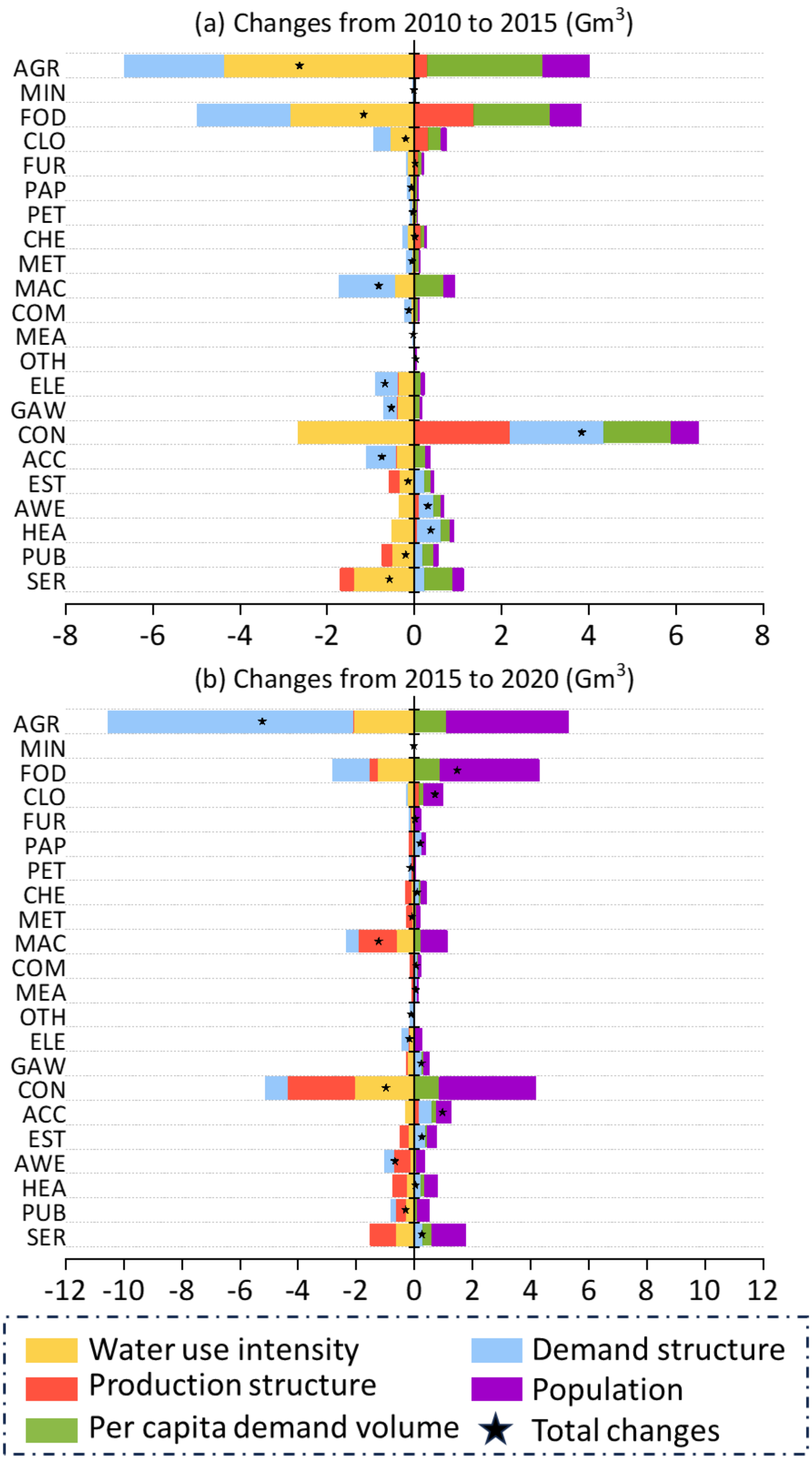

Figure S3. Contributions of different socioeconomic factors to water footprint changes at the sector level in the YRDUA over 2010-2020

Notes: Shanghai (SH), Nanjing (NJ), Wuxi (WX), Changzhou (CZ), Suzhou (SZ), Nantong (NT), Yancheng (YC), Yangzhou (YZ), Zhenjiang (ZJ), Taizhou in Jiangsu province (TZ),

Hangzhou (HZ), Ningbo (NB), Jiaxing (JX), Huzhou (HUZ), Shaoxing (SX), Jinhua (JH), Zhoushan (ZS), Taizhou in Zhejiang province (TAZ), Hefei (HF), Wuhu (WH), Ma'anshan (MAS), Tongling (TL), Anqing (AQ), Chuzhou (CUZ), Chizhou (CHZ), Xuancheng (XC).

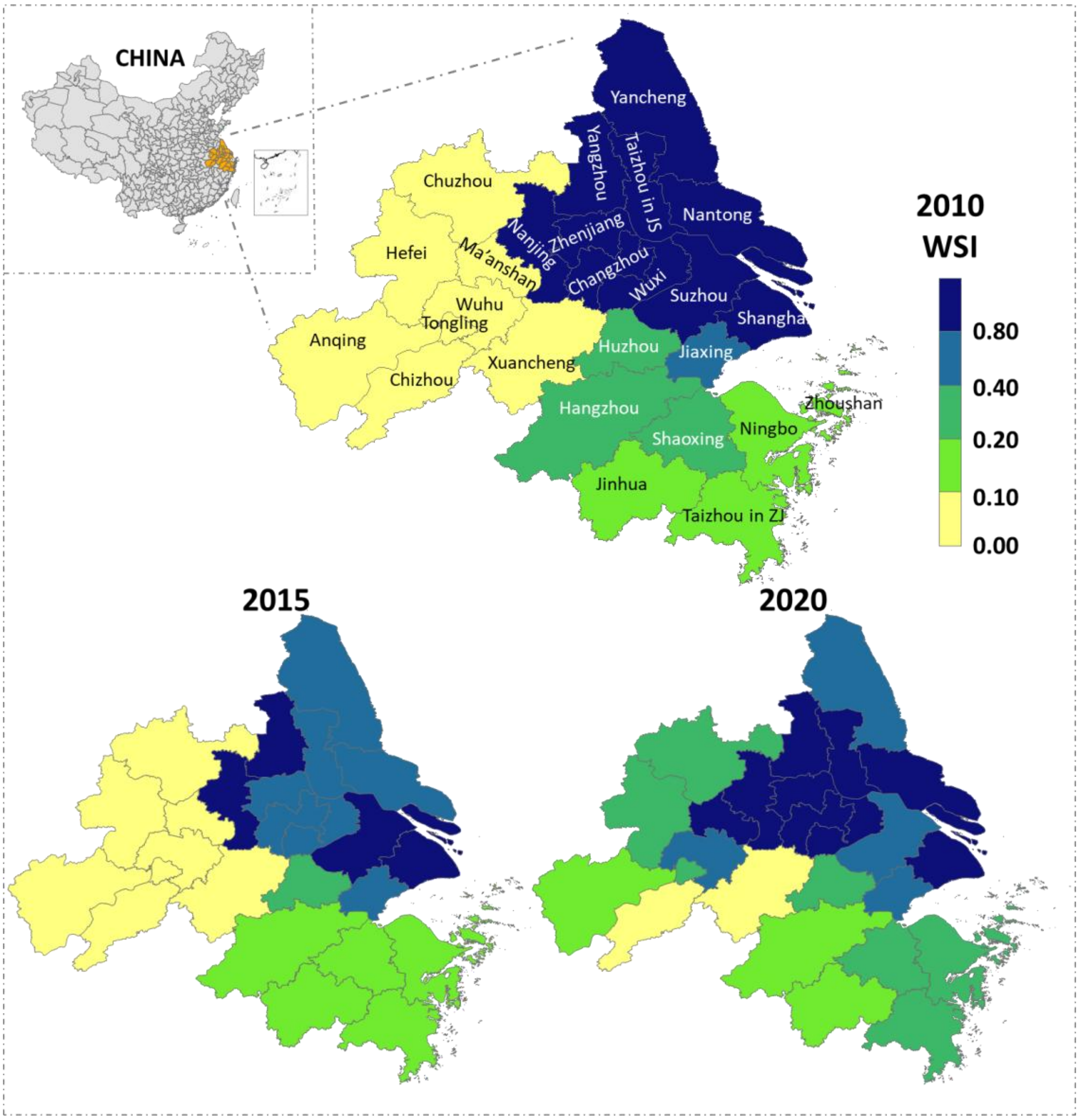

Figure S4. Changes of water stress index in the YRDUA over 2010–2020

Note: The WSI within the intervals of 0–0.1, 0.1–0.2, 0.2–0.4, 0.4–0.8 and >0.8 represent respectively low, low to medium, medium to high, high, and extremely high levels of water stress.

Supplementary Tables

Table S1. An overview of previous studies related to accounting of virtual water and water footprint.

Scale	Year	Study area	Methods	Research focus	Reference
National-level	1995-2009	Global	Input–output analysis and cluster analysis	Virtual water transfer pattens	[1]
	2012-2017	Global	Input–output analysis and water stress index	Water stress footprint patterns and international water stress flows	[2]
Provincial level	2017	China	Input–output analysis and virtual water inequality index	Spillover-feedback effects and inequalities in trade	[3]
	2012	China	Input–output analysis and water scarcity index	Virtual land-dependent water and non-land-dependent water transfers	[4]
	2002-2015	China	Input–output analysis and Logarithmic Mean Divisia Index models	Inter-regional virtual water transfers, and driving factors	[5]
	2002-2015	China	Input–output analysis and virtual	Virtual water transfers, and inequality	[6]

Scale	Year	Study area	Methods	Research focus	Reference
			water inequality index		
	2017	China	Input–output analysis and Structural Path Analysis	Transfer pattern of virtual scarce water	7
	2002-2017	China	Input–output analysis and Structural decomposition analysis	Temporal dynamics, driving factor and mutual relationship	8
	2012-2017	Yangtze River Economic Belt	Input–output analysis	Spatiotemporal pattern of virtual water	9
City-level	2012	Yangtze River Delta cities	Input–output analysis and water scarcity index	Spatial characteristics, and mitigation potential regarding virtual water trade on urban water scarcity	10
	2012, 2015	Beijing, Tianjin and Hebei capital agglomerations	Input–output analysis and spillover and feedback effects	The intra-regional multiplier effects and the inter-regional spillover and feedback effects	11
Basin-level	2007	Haihe River Basin	Input–output analysis and water scarcity index	Virtual water transfers, and mitigation potential regarding virtual water trade on urban water scarcity	12

Scale	Year	Study area	Methods	Research focus	Reference
County-level	2017	Yellow River Basin	Input–output analysis	Virtual water circulation patterns	[13]
	2017	California	Input–output analysis and index decomposition analysis	Virtual water transfers, and driving factors	[14]

Table S2. The aggregated and original sectors in the 2010 input-output table as an example.

Code	Aggregated sectors	Code	Original sectors
1	Agriculture (Agr)	1	Agriculture
2	Mining industry (Min)	2	Mining and washing of coal
		3	Mining and washing of petroleum and natural gas
		4	Mining and processing of metal ores
		5	Mining and processing of nonmetal ores and other ores
3	Manufacture of food and tobacco (FOD)	6	Manufacture of food and tobacco
4	Manufacture of clothing (CLO)	7	Manufacture of textiles
		8	Manufacture of textile wearing apparel, footwear, caps, leather, fur, feather and its products
5	Processing of timber and manufacture of furniture (FUR)	9	Processing of timber and manufacture of furniture
6	Papermaking, printing and manufacture of articles for culture, education and sports activities (PAP)	10	Papermaking, printing and manufacture of articles for culture, education and sports activities
7	Processing of refined petroleum, coking products, processing of nuclear fuel (PET)	11	Processing of refined petroleum, coking products, processing of nuclear fuel
8	Manufacture of chemicals and chemical products (CHE)	12	Manufacture of chemicals and chemical products
9	Manufacturing of metals (MET)	13	Manufacture of nonmetallic mineral products
		14	Manufacture of processing of metals

		15	Manufacture of fabricated metal products, expect machinery, and equipment
		16	Manufacture of general-purpose machinery
10	Manufacturing of machiner (MAC)	17	Manufacture of special-purpose machinery
		18	Manufacture of transport equipment
		19	Manufacture of electrical machinery and apparatus
11	Manufacture of communication equipment, computer, and other electronic equipment (COM)	20	Manufacture of communication equipment, computer, and other electronic equipment
12	Manufacturing of measuring instruments (MEA)	21	Manufacture of measuring instruments
13	Other manufacture (OTH)	22	Other manufacture
12	Manufacturing of measuring instruments (MEA)	23	Scrap and waste
		24	Repair of fabricated metal products, machinery, and equipment
14	Production and supply of electricity and steam (ELE)	25	Production and supply of electricity and steam
15	Production of gas and water (GAW)	26	Production and distribution of gas
		27	Production and distribution of water
16	Construction (CON)	28	Construction
22	Other services (SER)	29	Transport, storage, and post
		30	Wholesale and retail trade
17	Accommodation, food, and beverage services (ACC)	31	Accommodation, food, and beverage services
22	Other services (SER)	32	Information transmission, software,

			and information technology services
		33	Finance
18	Real estate (EST)	34	Real estate
22	Other services (SER)	35	Renting and leasing, business services
		36	Scientific research and development, technical services
19	Management of water conservancy, environment and public facilities (AWE)	37	Management of water conservancy, environment and public facilities
22	Other services (SER)	38	Services to households, repair and other services
		39	Education
20	Healthcare and social work activities (HEA)	40	Healthcare and social work activities
22	Other services (SER)	41	Culture, sports, and entertainment
21	Public management, social security, and social organization (PUB)	42	Public management, social security, and social organization
22	Other services (SER)	38	Services to households, repair and other services

Supplementary references

1. Tian, X., Sarkis, J., Geng, Y., Qian, Y., Gao, C., Bleischwitz, R., and Xu, Y. (2018). Evolution of China's water footprint and virtual water trade: A global trade assessment. Environ. Int. *121*, 178-188. https://doi.org/10.1016/j.envint.2018.09.011.
2. Zhao, H., Miller, T.R., Ishii, N., and Kawasaki, A. (2022). Global spatio-temporal change assessment in interregional water stress footprint in China by a high resolution MRIO model. Sci. Total Environ. *841*, 156682. https://doi.org/10.1016/j.scitotenv.2022.156682.
3. Wei, Z., Huang, K., Chen, Y., Wang, D., Yu, Y., Xu, M., and Kapelan, Z. (2023). Unveiling the inequalities in virtual water transfer in China: The environmental and economic perspectives. Sustain. Prod. Consump. *42*, 63-73. https://doi.org/10.1016/j.spc.2023.09.009.
4. Tian, P., Lu, H., Liu, J., Feng, K., Heijungs, R., Li, D., and Fan, X. (2022). The pattern of virtual water transfer in China: From the perspective of the virtual water hypothesis. J. Clean Prod. *346*, 131232. https://doi.org/10.1016/j.jclepro.2022.131232.
5. Zhong, Z., Chen, Z., and Deng, X. (2022). Dynamic change of inter-regional virtual water transfers in China: Driving factors and economic benefits. Water Resour. Econ. *39*, 100203. https://doi.org/10.1016/j.wre.2022.100203.
6. Xin, M., Wang, J., and Xing, Z. (2022). Decline of virtual water inequality in China's inter-provincial trade: An environmental economic trade-off analysis. Sci. Total Environ. *806*, 150524. https://doi.org/10.1016/j.scitotenv.2021.150524.
7. Xu, Z., Xu, C., Chen, X., Yang, L., and Song, M. (2024). Interprovincial industrial virtual scarce water flow and water scarcity risk in China. Ecol. Indic. *169*, 112939. https://doi.org/10.1016/j.ecolind.2024.112939.
8. Wang, H., Yang, X., Hou, H., Liu, T., Zhang, Y., and Xu, H. (2023). Temporal dynamics, driving factor and mutual relationship analysis for the holistic virtual water trade network in China (2002–2017). Environmental Impact Assessment Review *101*, 107127. https://doi.org/10.1016/j.eiar.2023.107127.
9. Yang, Y., Tian, G., Li, J., and Sun, Z. (2024). Evolution of spatiotemporal pattern of

virtual water in the Yangtze River economic belt. Ecol. Indic. *161*, 111967. https://doi.org/10.1016/j.ecolind.2024.111967.

10. Zhu, M., Wang, J., Zhang, J., and Xing, Z. (2022). The impact of virtual water trade on urban water scarcity: A nested MRIO analysis of Yangtze River Delta cities in China. J. Clean Prod. *381*, 135165. https://doi.org/10.1016/j.jclepro.2022.135165.
11. Chen, Y., Huang, K., Hu, J., Yu, Y., Wu, L., and Hu, T. (2021). Understanding the two-way virtual water transfer in urban agglomeration: A new perspective from spillover-feedback effects. J. Clean Prod. *310*, 127495. https://doi.org/10.1016/j.jclepro.2021.127495.
12. White, D.J., Feng, K., Sun, L., and Hubacek, K. (2015). A hydro-economic MRIO analysis of the Haihe River Basin's water footprint and water stress. Ecol. Model. *318*, 157-167. https://doi.org/10.1016/j.ecolmodel.2015.01.017.
13. Yang, H., Wang, Y., Peng, B., Zhang, X., and Zou, H. (2024). Re-examining virtual water transfer in the Yellow River Basin, China. Journal of Hydrology: Regional Studies *56*, 101971. https://doi.org/10.1016/j.ejrh.2024.101971.
14. Liu, B., Feng, K., Sun, L., Baiocchi, G., Wang, D., and Miralles-Wilhelm, F. (2025). Assessing virtual water trade and inequalities in household water footprints across California's counties. Struct. Change and Econ. Dyn. *74*, 175-185. https://doi.org/10.1016/j.strueco.2025.02.008.
